# Gut microbiota are associated with sex and age of host: Evidence from semi‐provisioned rhesus macaques in southwest Guangxi, China

**DOI:** 10.1002/ece3.7643

**Published:** 2021-05-14

**Authors:** Yuhui Li, Ting Chen, Youbang Li, Yin Tang, Zhonghao Huang

**Affiliations:** ^1^ Guangxi Key Laboratory of Rare and Endangered Animal Ecology Guangxi Normal University Guilin China; ^2^ College of Life Sciences Guangxi Normal University Guilin China; ^3^ College of Arts Guilin University of Technology Guilin China

**Keywords:** age difference, gut microbiota, *Macaca mulatta*, sex difference

## Abstract

Host characteristics, such as sex and age, are closely associated with the structure and function of gut microbiota; however, less is known about the effects of age and sex on the gut microbiota of nonhuman primates, and therefore, our knowledge of interindividual variability in host gut microbiota is limited. In this study, 153 fecal samples from rhesus macaques (*Macaca mulatta*) were analyzed using high‐throughput 16S rRNA sequencing in order to explore associations between age and sex of the host and their gut microbiota. The results indicated that female macaques had higher alpha diversity and a more unique gut microbiota than did males. The proportion of Proteobacteria, Tenericutes, Cyanobacteria, unclassified bacteria, and Verrucomicrobia was higher in females than that in males. We also found that adults of both sexes had a higher alpha diversity, a higher proportion of norank *Ruminococcaceae*, *Oscillospira*, norank *Lachnospiraceae*, norank Clostridiales, and *Succinivibrio,* and a lower proportion of *Enterococcus* than immatures. Functional analyses revealed that the richness of metabolic pathways was higher in females than males and in adults compared with immatures. These results could be attributed to differences in the nutritional requirements and hormone levels of macaques of different sex and age classes. We conclude that variation in the gut microbiota of different sex and age classes of rhesus macaques may be linked to age‐ and sex‐specific differences in nutrient requirements and hormone levels. These results highlight the importance of host age and sex on the structure and function of the gut microbiota and the need to consider physiological traits when conducting studies on the gut microbiota.

## Graphical abstract

Sex and age were associated with differences in the diversity and relative abundance of gut microbiota in rhesus macaques. These differences could be linked to age‐ and sex‐specific differences in nutrient requirements and hormone levels, highlighting the effects of age and sex on the structure and function of the gut microbiota, as well as the need to consider physiological traits when conducting gut microbiota studies.

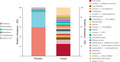

## 1 INTRODUCTION

Interindividual differences in sex and age strongly affect host gut microbiota (Derrien et al., 2019; Koren et al., 2012; Markle et al., 2013; Peng et al., 2020; Reveles et al., 2019; Vujkovic‐Cvijin et al., 2020). Numerous studies have focused on the effect of diet on gut microbiota. These studies indicate that diet can rapidly alter the composition of a host's gut microbiota (David et al., 2014; De Filippo et al., 2010). Different diets provide a variety of nutritional substrates to the intestinal microbiota, allowing its growth and reproduction (David et al., 2014; Ley et al., 2008). However, the importance of sex and age on hosts' gut microbiota has generally been overlooked (Amato et al., 2014; Kashtanova et al., 2016). There are studies that indicate that females tend to increase their consumption of nutrient‐rich foods during pregnancy and lactation (Koch et al., 2017), and mammalian diets tend to change from breastmilk to complex foods during the growth process (Kashtanova et al., 2016). Therefore, individuals of different age and sex classes are likely to exhibit differences in their gut microbiota (Byrd et al., 2020).

Sex differences in mammalian behavior and physiology are extremely common, which may further cause changes in their gut microbiota (Dunbar et al., 2009; Koch et al., 2017; Koren et al., 2012; O'Mara & Hickey, 2014). Previous studies have demonstrated that gut microbiota diversity in females is higher than in males, which could be associated with sex‐based differences in diet (Amato et al., 2014; de la Cuesta‐Zuluaga et al., 2019). In Verreaux's sifakas (*Propithecus verreauxi*), for example, females were found to devote more time to feeding and increased the intake of macronutrients than males (Koch et al., 2017). In ring‐tailed lemurs (*Lemur catta*), females altered their diet in order to compensation for higher nutritional and/or energetic demands caused by reproduction (Dunbar et al., 2009; Koch et al., 2017; Li et al., 2014; O'Mara & Hickey, 2014).

Based on these sex‐based differences in diet, the mammalian gut microbiota might be subjected to different selective pressures, further promoting the formation of dimorphism in gut microbiota (David et al., 2014). Conversely, although males and females living in the same social group generally share the same dietary pattern, their microbiota may be affected by differences in the timing and production of sex steroids (Markle et al., 2013; Peng et al., 2020; vom Steeg & Klein, 2017; Yurkovetskiy et al., 2013). Researchers have confirmed that gut microbiota can regulate or metabolize testosterone and estrogen and employ sex steroids for growth and survival (Baker et al., 2017; vom Steeg & Klein, 2017). For instance, rodent disease models indicated that the gut microbiota of males was more susceptible to the development of metabolic disorders than that of females (Peng et al., 2020). This occurred because the males were exposed to a high‐fat diet (Peng et al., 2020). However, the testosterone levels of female recipient increase and metabolic changes when the gut microbiota of adult males are transferred to the intestines of immature females, which results in the prevalence of islet inflammation decreased significantly (Markle et al., 2013). These differences were attributed to sex steroids (Markle et al., 2013; Peng et al., 2020). Thus, considering both nutritional requirements and hormonal differences, the interaction between sex and gut microbiota needs to be carefully studied (vom Steeg & Klein, 2017).

Age is also associated with the gut microbiota of mammals (Byrd et al., 2020; Hasegawa et al., 2018). This has been documented in humans (de la Cuesta‐Zuluaga et al., 2019) and nonhuman primates, such as wild western lowland gorillas (*Gorilla gorilla gorilla*) (Pafčo et al., 2019), black howler monkeys (*Alouatta pigra*) (Amato et al., 2014), and Tibetan macaques (*Macaca thibetana*) (Sun et al., 2018). In general, the diversity of the gut microbiota increases with the host age and stabilizes after reaching an adult‐like microecosystem and digestive ability (Derrien et al., 2019; Kashtanova et al., 2016; But see Reese et al., 2021). This trend may be related to the complexity of dietary composition at different growth stages. Dietary complexity in adults is higher than that in juveniles and infants who have limited mobility or hunting skills (Schiel et al., 2010). In addition, different age classes are differentially affected by hormones, especially during puberty (Vandenberg et al., 2012). Therefore, studies of the gut microbiota need to consider the effects of age on digestive ecology (Byrd et al., 2020; Hasegawa et al., 2018).

Rhesus macaques (*Macaca mulatta*) are frequently used as animal models to explore interactions between the gut microbiota and diseases (Cui et al., 2019). Previous studies have shown that the dominant genus in the gut microbiota of rhesus macaques was *Prevotella*, followed by norank *Ruminococcaceae*, and norank *Clostridiaceae* (Chen et al., 2020a). These gut microorganisms are specialists in digesting cellulose (De Filippo et al., 2010; Koeck et al., 2014; La Reau & Suen, 2018). In Tibetan macaques, the abundance of *Ruminococcaceae* increased following the increase in cellulose and lignin content in their diet (Zhao et al., 2018). In addition to diet, factors that influence the gut microbiota of rhesus macaque include interspecific differences in physiology (Chen et al., 2020b; Cui et al., 2019) and environment (Chen et al., 2020a; Zhao et al., 2018). While age and sex are known to influence gut microbiota composition (Byrd et al., 2020; de la Cuesta‐Zuluaga et al., 2019; Hasegawa et al., 2018), more research is needed on this relationship in nonhuman primates. Particularly for species used as animal models, disregarding sex and/or age information may exacerbate the risk of obtaining false positives in gut microbiota studies of human disease (Vujkovic‐Cvijin et al., 2020). For example, although *Streptococcus gordonii* may be associated with increased risk of infective endocarditis (Douglas et al., 1993), a recent cohort study suggests that it is also strongly related to age and may even serve as a gut microbial marker for predicting host age (Zhang et al., 2021). Therefore, we explored associations between sex and age and the gut microbiota of rhesus macaques. In this study, 153 fecal samples from one group of semi‐provisioned rhesus macaques were collected and differences in the composition, abundance, diversity, and function of the gut microbiota were analyzed by sex and age. We tested the following predictions:

1. Considering the higher nutritional and metabolic requirements caused by reproduction (Amato et al., 2014; Dunbar et al., 2009), adult females are expected to have higher diversity and richness in their gut microbiota than adult males;
2. Considering the higher dietary complexity in adults (Schiel et al., 2010), *Ruminococcaceae* and *Clostridiaceae*, which are typically associated with fiber metabolism, are expected to be in higher abundance in adult rhesus macaques than in immatures.

## 2 METHODS

### 2.1 Study site and subjects

This study was conducted in the Guangxi Longhu Mountain Nature Reserve in Long'an County, Nanning, Guangxi, China (22°56′–23°00′N, 107°27′–107°41′E). This natural reserve is a scenic spot, covered by karst landforms with an altitude of approximately 300–500 m above sea level and dominated by subtropical mountain monsoon rain forest (Wang et al., 1996). The study area has a tropical monsoon climate, with an annual mean temperature of 21.8°C, and an annual mean rainfall of 1,500 mm (Wang et al., 1996).

The study subjects were members of a group comprising approximately 400 rhesus macaques. Their diet was comprised of cooked corn provided by the park managers twice daily (10 a.m. and 3 p.m.) and peanuts provided by visitors. During nonprovisioned time or when visitors were not present, they mainly relied on natural food resources, such as leaves and fruits (Chen et al., 2020a).

### 2.2 Sample collection

We collected fecal samples of rhesus macaques using sterile gloves and used sterilized bamboo sticks to remove the outer layer of feces that had been in contact with the ground. The inner part of the stool was placed into a sterile centrifuge tube and put into a dry ice portable case to be sent to the laboratory for storage at −80°C until DNA was extracted. In total, 153 rhesus macaque fecal samples were collected (Table 1).

**TABLE 1:** Host information on 153 fecal samples of rhesus macaques

Month	No. of samples	Sex		Age	
		Male	Female	Adult	Immature
October 2018	7	2	2	5	2
November 2018	1		1	1	
December 2018	6	1	3	6	
January 2019	6			4	2
February 2019	13	4	3	3	10
March 2019	12	3	9	10	2
April 2019	11	6	5	7	4
May. 2019	12	8	4	5	7
June 2019	12	3	9	10	2
July 2019	12	4	7	7	5
August 2019	13	5	7	10	3
September 2019	12	8	4	7	5
October 2019	12	3	9	12	
November 2019	12		12	10	2
December 2019	12	2	10	12	
Total	153	49	85	109	44

All samples were categorized by sex (male or female) and age (adult or immature). A total of 109 samples were adult (≥ 5 years old), and 44 samples were immature (< 5 years old). Sex was identified in 134 samples and unidentified in 19 samples. Of the 134 identified samples, 49 were males and 85 were females. Samples for which sex was not determined were excluded from subsequent analysis.

### 2.3 DNA extraction, 16S rRNA gene amplification, and sequence processing

The total DNA of gut microbiota from fecal samples was extracted using the E.Z.N.A. Soil DNA Kit (Omega Bio‐Tek) following the manufacturer's protocol; next, the concentration of the extracted DNA was tested and purified using a NanoDrop 2000 ultraviolet‐visible light spectrophotometer (Thermo Fisher Scientific, Inc), followed by 1% agarose gel electrophoresis. Bacterial primers 338F (5′‐ACTCCTACGGGAGGCAGCAG‐3′) and 806R (5′‐GGACTACHVGGGTWTCTAAT‐3′) targeting the V3–V4 hypervariable region of the 16S rRNA gene were used for polymerase chain reaction (PCR) amplification. The PCR products were extracted from a 2% agarose gel and purified further with the AxyPrep DNA Gel Extraction Kit (Axygen Biosciences) and quantified using the QuantiFluor™‐ST (Promega) following the manufacturer's protocol. Purified amplicons were sequenced on an Illumina MiSeq platform at Majorbio Bio‐Pharm Technology Co. Ltd.

The 16S rRNA raw reads obtained from MiSeq sequencing were filtered to obtain high‐quality sequences using FLASH (version 1.2.11; https://ccb.jhu.edu/software/FLASH/index.shtml) as follows: (a) We set up a 50 bp window. Next, the sequence with an average quality score of < 20 from the sliding window was truncated, and any sequence of < 50 bp during quality control was removed; (b) barcodes were matched exactly. The barcode and primer sequences at both ends of the sequence were used to distinguish samples to obtain effective sequences. The direction of sequences was corrected, and the ambiguous bases were discarded. The maximum mismatch number of barcodes and primers was zero and two, respectively; (c) sequences were stitched. Sequences with overlap of > 10 bp were merged according to overlapping relationship of paired‐end reads. The sequences that could not be stitched were removed (Bokulich et al., 2013; Caporaso et al., 2010).

### 2.4 Bioinformatics analysis

The high‐quality sequences were clustered into operational taxonomic units (OTUs) with a 97% similarity using UPARSE (version 7.0.1090; http://drive5.com/uparse) (Edgar, 2013), and the chimeric sequences were removed using UCHIME.37 (Edgar et al., 2011). Species taxonomic analysis of OTU was performed by the ribosomal database program classifier Bayesian algorithm (version 2.11; https://sourceforge.net/projects/rdp‐classifier/) and aligned to the Greengenes 16S bacteria database (version 13.5), with a confidence threshold of 80%. Good's coverage scores were calculated to determine species coverage using the Mothur software (version 1.30.1; http://www.mothur.org/wiki/Schloss_SOP#Alpha_diversity) (Schloss et al., 2009). Rarefaction curves were generated using the R statistical software for each sample to assess sequencing depth. The relative abundance of community composition was represented by mean ± standard deviation (*SD*). The differences in relative abundance within sex and age categories were analyzed using the Wilcoxon rank‐sum test. A false discovery rate was used to correct *p*‐values. Linear discriminant analysis (LDA) and effect size (LEfSe) (http://huttenhower.sph.harvard.edu/galaxy/) were conducted to further detect taxa (from phylum to genus) with differential abundance by sex and age categories (LDA score ≥3; a significance of *p* < 0.05 was determined using the nonparametric factorial Kruskal–Wallis sum‐rank test).

Alpha diversity was assessed using two diversity indices (Shannon and Simpson) and two richness indices (Ace and Chao), calculated using Mothur software, and visualized in R (version 4.0.4) (R Core Team, 2021). A lower Simpson index suggested a higher alpha diversity. We examined the effect of interaction between age and sex on alpha diversity using *lmer* and *anova* functions in lme4 package of R (Bates et al., 2015). We set alpha diversity as a response variable and age, sex, and age–sex interaction as fixed factors and include sampling time as a random factor. For the purpose of improving linearity, the Shannon index, ACE, and Chao values were log_10_(*X*)‐transformed and the Simpson index was logit(*X*)‐transformed (Li et al., 2020; Warton & Hui, 2011). We ran likelihood tests comparing general linear mixed models with and without the age–sex interaction. The results suggested a significant influence of age–sex interaction on the alpha diversity when the *p*‐value was <0.05. Our results showed that there were no significant effects of age/sex interaction on the alpha diversity (Shannon index: *χ* ^2^ = 0.234, *df* = 2, *p* = 0.890; Simpson: *χ* ^2^ = 0.429, *df* = 2, *p* = 0.807; Ace: *χ* ^2^ = 1.349, *df* = 2, *p* = 0.510; Chao: *χ* ^2^ = 1.497, *df* = 2, *p* = 0.473). Thus, the Wilcoxon rank‐sum test was used to analyze alpha diversity differences between sex and age groups. To assess differences in community composition and structure, principal coordinate analysis (PCoA) and permutational multivariate analysis of variance (PERMANOVA) were performed based on the Bray–Curtis dissimilarity matrix calculated by QIIME (version 1.9.1; http://qiime.org/install/index.html).

The functional profiles from the 16S rRNA data were predicted using the Phylogenetic Investigation of Communities by Reconstruction of Unobserved States 2 program (PICRUSt2) (version 2.1.3‐b; http://picrust.github.io/picrust/) to better understand the bacterial functional profiles associated with sex and age. The Nearest Sequenced Taxon Index (NSTI) was calculated for evaluating accuracy of functional profiles, with a lower value indicating a higher prediction accuracy (Langille et al., 2013). The mean NSTI for all samples was 0.11 ± 0.03, which was lower than the mean NSTI of mammals (0.14 ± 0.06) (Langille et al., 2013). Predictive functional pathways were annotated using the Kyoto Encyclopedia of Genes and Genomes (KEGG) (http://www.genome.ad.jp/kegg/) at level 1 to level 3 KEGG orthology groups (KOs). Mann–Whitney U test was used to examine differences in metabolic pathways in level 3 between two groups (male vs. female; adult vs. immature). Significant differences were set at *p* < 0.05.

## 3 RESULTS

### 3.1 Summary of sequencing data

**FIGURE 1:**
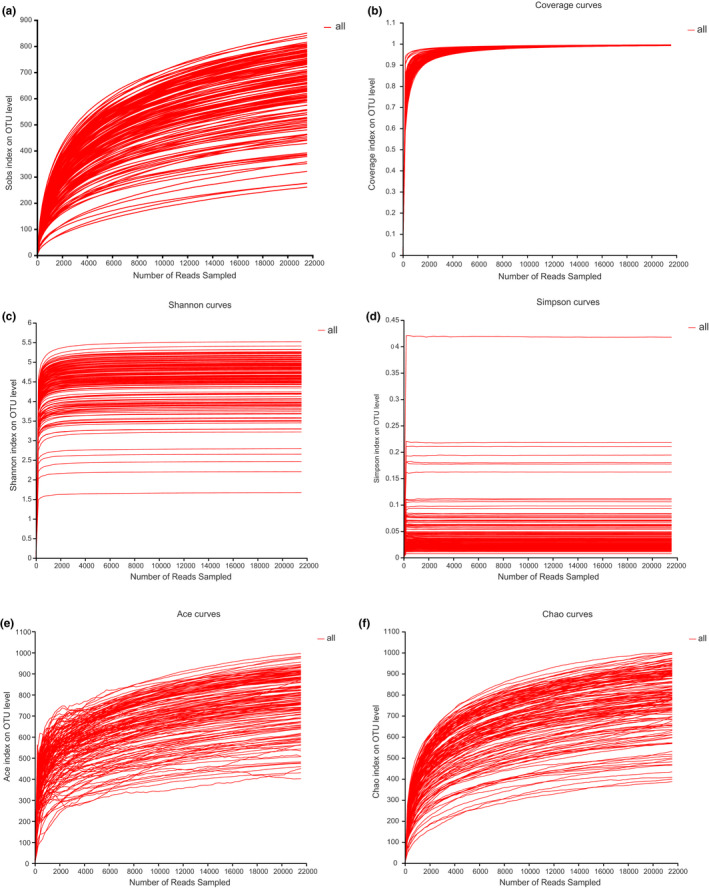
Rarefaction curves, coverage, and results of Shannon, Simpson, Ace, and Chao tests

After quality filtering and removal of the chimera, a total of 7,691,681 effective reads were obtained from 153 fecal samples, with an average of 50,272 ± 9,265 effective reads, an average length of 61,847 ± 8,521 effective tags, and an average length of 416 ± 6 bp. The Good's coverage estimates of the 153 samples ranged from 99.12% to 99.61% (mean ± *SD* = 99.28% ± 0.11%), suggesting that almost all bacterial taxa in each sample were identified. The end of the rarefaction curve tended to be an asymptote, indicating that the sample size in this study was sufficient (Figure 1). After subsampling all samples to an equal sequencing depth (21,561 reads per sample) and clustering, 1,770 OTUs at 97% sequence similarity were obtained and classified into 26 phyla, 59 classes, 103 orders, 180 families, and 298 genera.

### 3.2 Differences in the composition and abundance of gut microbiota

**FIGURE 2:**
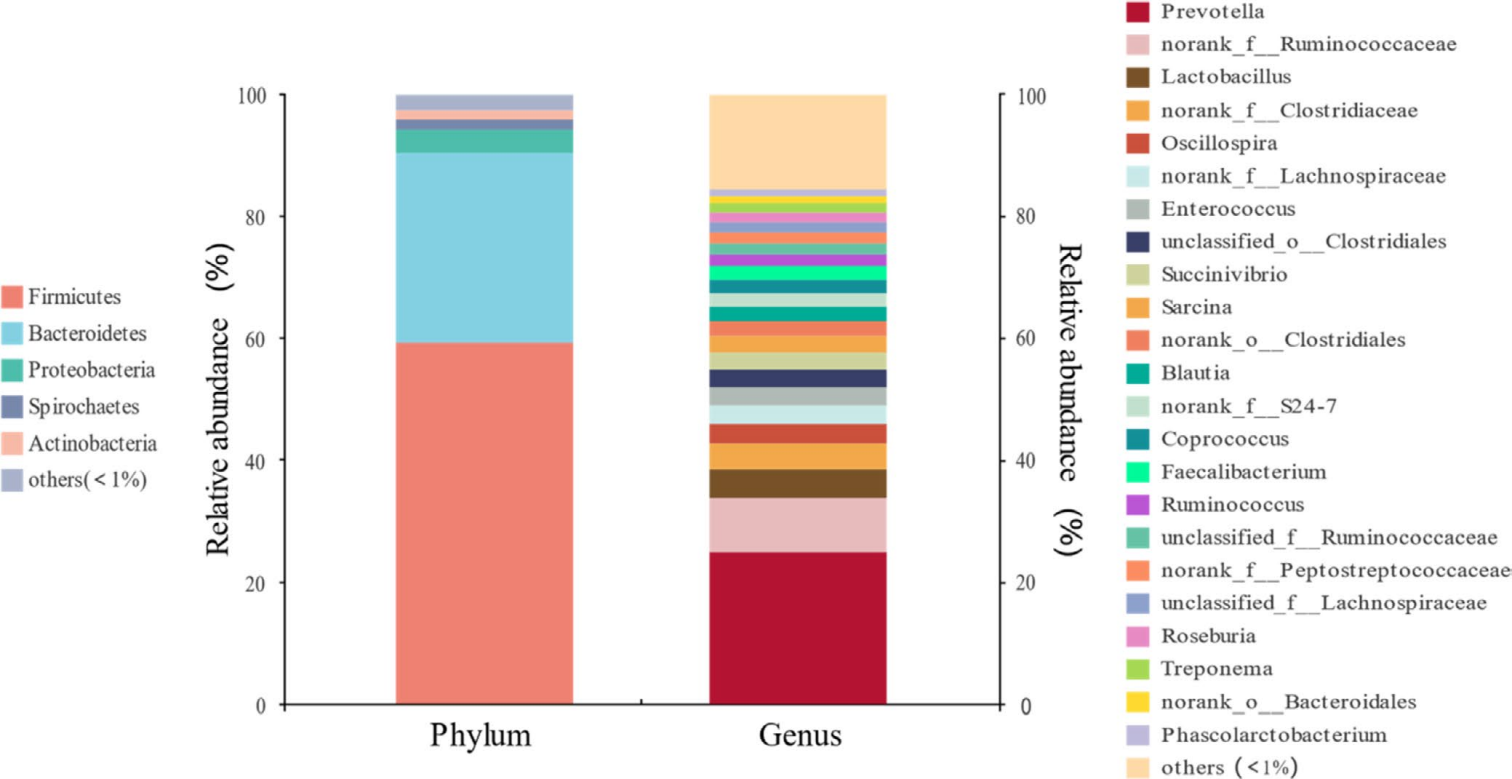
Gut microbiota composition at the level of phylum and genus

Based on all samples, the three phyla with the highest relative abundance were Firmicutes (59.31% ± 16.76%), Bacteroidetes (31.11% ± 16.31%), and Proteobacteria (3.85% ± 4.81%). The top three genera with the highest realative abundance were *Prevotella* (24.98% ± 15.98%), norank *Ruminococcaceae* (8.84% ± 4.65%), and *Lactobacillus* (4.75% ± 8.93%) (Figure 2).

At the phylum level, considering both sex and age, the three dominating phyla were Firmicutes, Bacteroidetes, and Proteobacteria (see Table A1). At the genus level, *Prevotella* had the highest relative abundance in both sex and age classes, followed by norank *Ruminococcaceae*, *Lactobacillus*, and norank *Clostridiaceae* (Tables A2, A3 and A2, A3).

**FIGURE 3:**
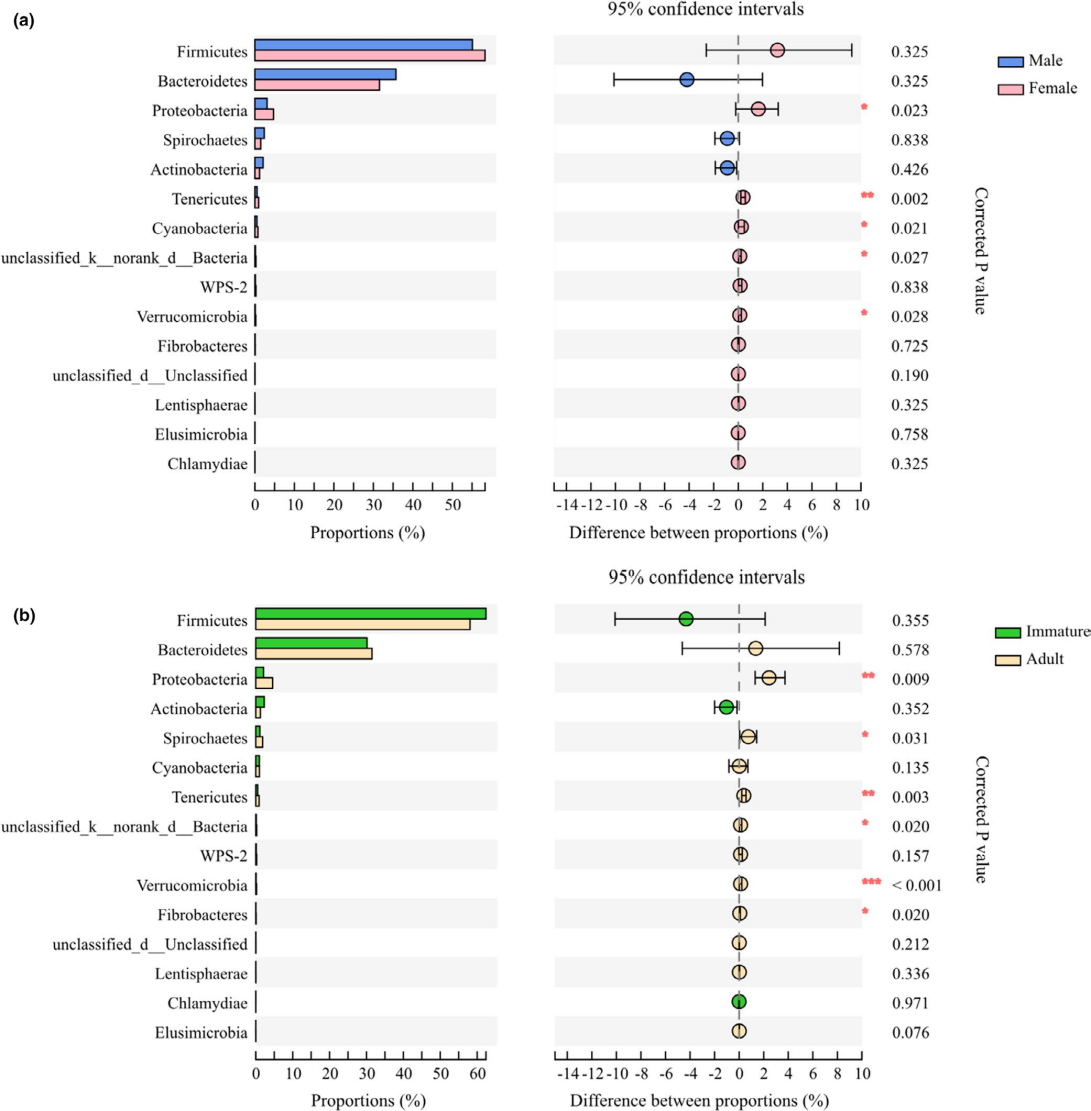
The gut microbiota composition and difference at the phylum level in sex (a) and age (b). The *p*‐value is represented by asterisks. Significant difference 0.01 < *p* < 0.05 is marked as “*”, 0.001 < *p* < 0.01 is marked as “**”, and *p* < 0.001 is marked as “***”

In analyzing the samples by sex, 22 bacterial phyla were detected in the male group and 24 phyla were detected in the female group. At the phylum level, Synergistetes and WS6 were found only in females. Among the top 15 phyla based on relative abundance, the proportions of Proteobacteria, Tenericutes, Cyanobacteria, unclassified bacteria, and Verrucomicrobia were higher in females (Figure 3a). At the genus level, male rhesus macaques had 17 unique genera and females had 40 unique genera. No significant differences were observed between males and females in the top 15 genera (Table A2).

In evaluating age‐based differences, 23 bacterial phyla were detected in the immature group and 26 phyla were detected in adults. Synergistetes, WS6, and norank Bacteroidetes were exclusively found in the adult group. Among the top 15 phyla, the proportion of Proteobacteria, Spirochaetes, Tenericutes, unclassified bacteria, Verrucomicrobia, and Fibrobacteres were higher in adults than that in immatures (Figure 3b). At the genus level, immatures had 36 unique genera and adults had 31 unique genera. Among the top 15 genera, adults had a higher proportion of norank *Ruminococcaceae*, *Oscillospira*, norank *Lachnospiraceae*, norank Clostridiales, and *Succinivibrio*, and a lower proportion of Enterococcus than did immatures (Table A3).

**FIGURE 4:**
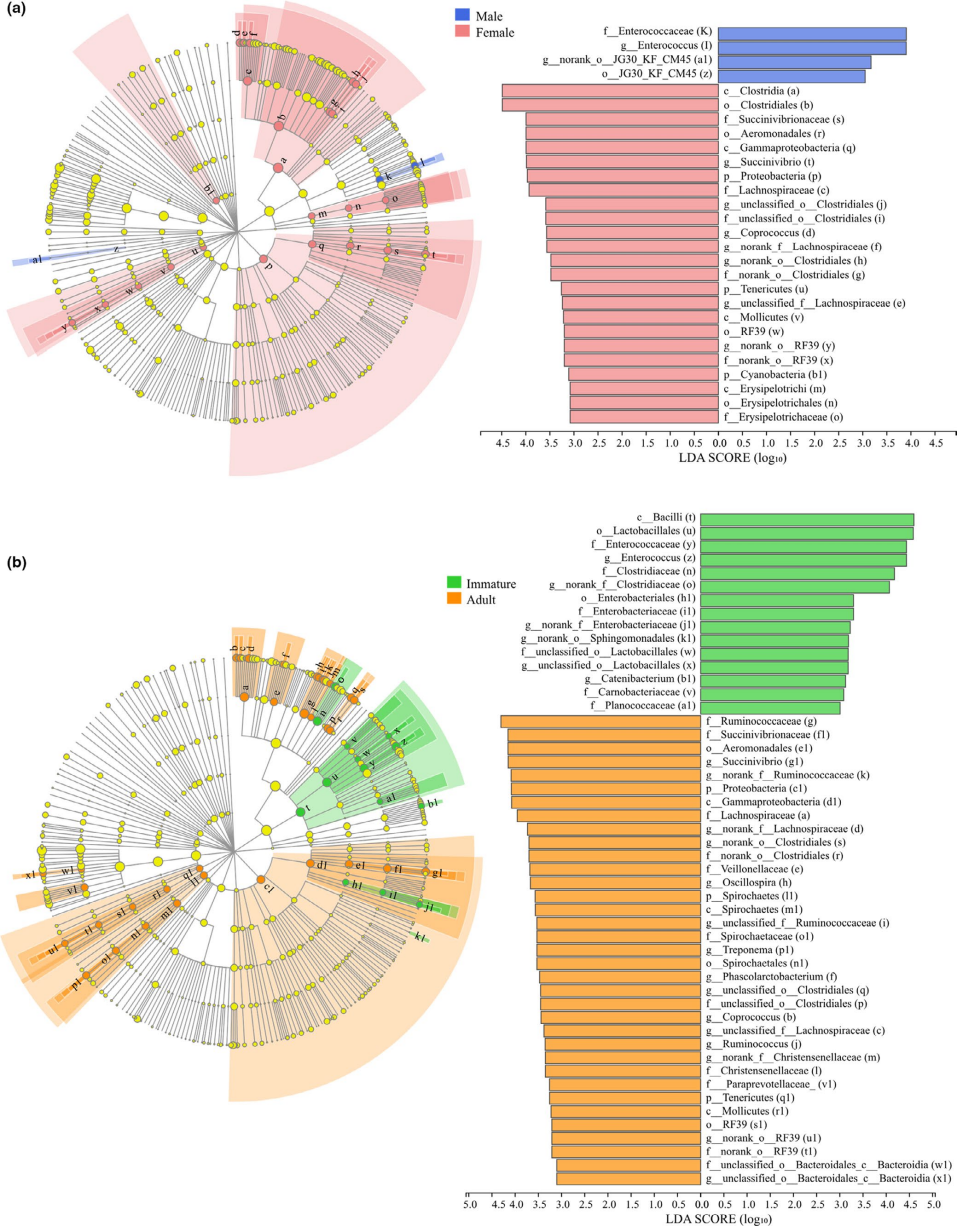
The LEfSe of gut microbiota abundance of semi‐provision rhesus macaques in sex (a) and age (b). Cladogram showing the relationship among taxa (from the inner to outer rings, phylum, class, order, family, and genus). The bar plot showing the different taxa with a LDA score of > 3

LEfSe analysis showed that 28 gut bacterial taxa were significantly different between males and females, of which four taxa were from the male group and 24 were from the female group. The class Clostridia, the order Clostridiales, and the family Succinivibrionaceae were the major taxa contributing to sex differences (Figure 4a). In the age category, 50 gut bacterial taxa were significantly different between adults and immatures, of which 35 were from the adult group and 15 taxa were from the immature group. The class Bacilli, the order Lactobacillales, the family *Enterococcaceae*, and the family *Ruminococcaceae* were the most important taxa contributing to age differences (Figure 4b).

### 3.3 Difference in diversity of gut microbiota

**FIGURE 5:**
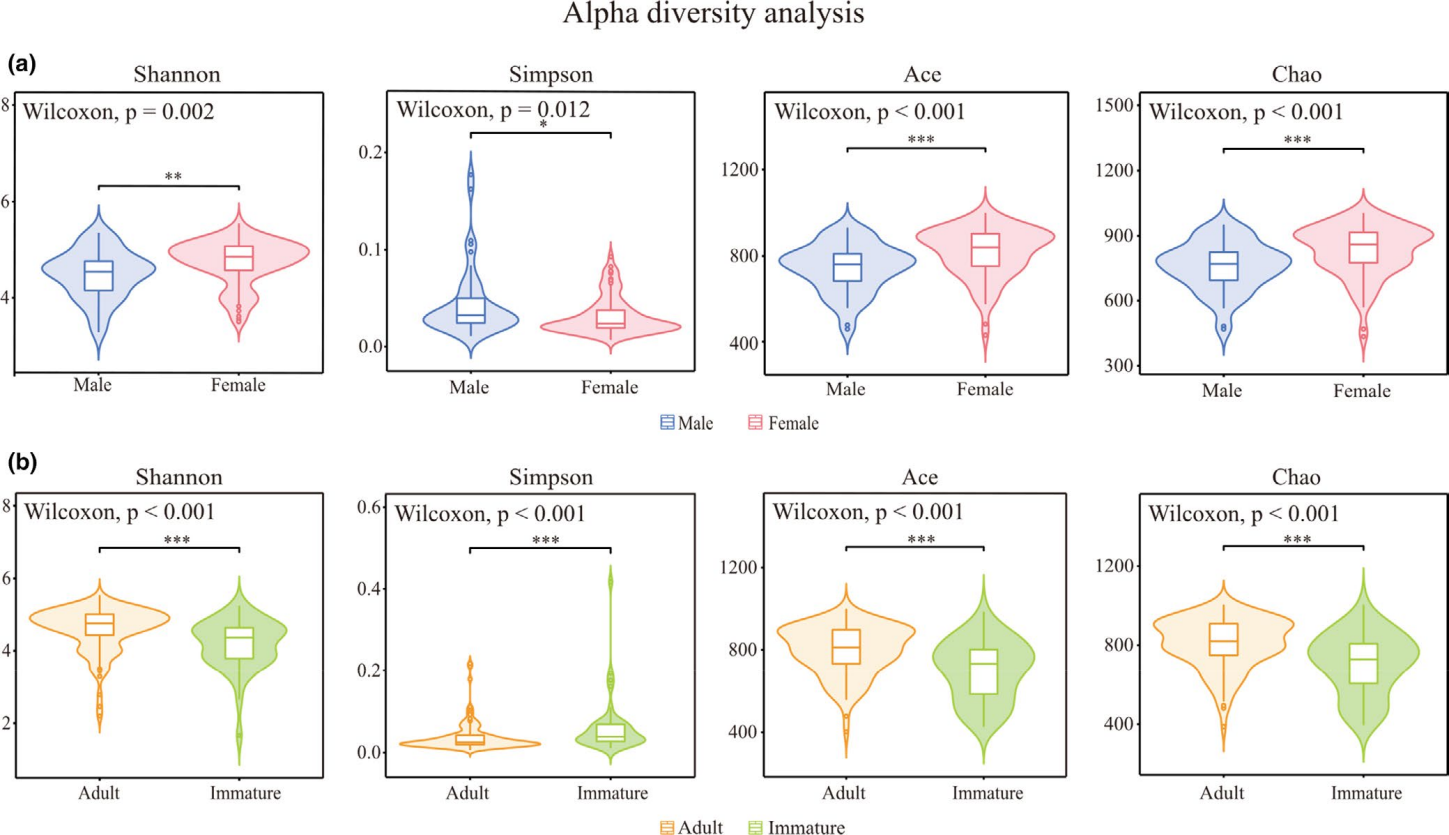
Alpha diversity difference in gut microbiota within sex (a) and age (b). The *p*‐value is represented by asterisks. Significant difference 0.01 < *p* < 0.05 is marked as “*”, 0.001 < *p* < 0.01 is marked as “**”, and *p* < 0.001 is marked as “***”

**FIGURE 6:**
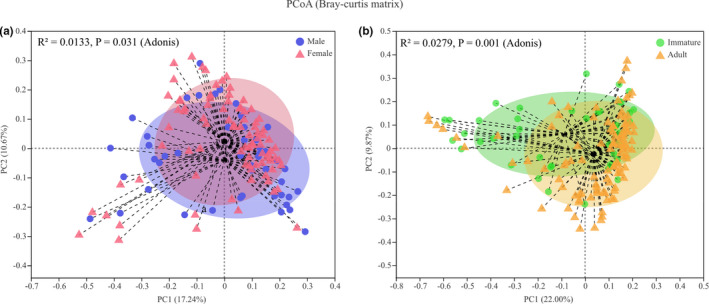
Beta diversity difference in gut microbiota within sex (a) and age (b). The ellipses represent 95% confidence intervals in multivariate space. The dotted lines represent the distance of every sample to the group's centroid

Alpha diversity indices were significantly different among sex–age classes. Females had higher Shannon, Ace, and Chao indices and a lower Simpson index than did males. The adult group had higher Shannon, Ace, and Chao indices and a lower Simpson index than did immatures (Figure 5 and Table 2). PCoA based on Bray–Curtis distances showed a significant difference in the community structure of the gut microbiota among sex–age classes (sex: *R* ^2^ = 0.0133, *p* = 0.031; age: *R* ^2^ = 0.0279, *p* = 0.001; Figure 6).

**TABLE 2:** Alpha diversity of the gut microbiota in rhesus macaques

Estimators	Male	Female	*p*‐value (sex)	Adult	Immature	*p*‐value (age)
Shannon	4.439 ± 0.507	4.703 ± 0.473	0.002	4.608 ± 0.605	4.176 ± 0.682	<0.001
Simpson	0.044 ± 0.035	0.032 ± 0.019	0.012	0.038 ± 0.035	0.061 ± 0.068	<0.001
Ace	743.510 ± 112.402	816.678 ± 115.477	<0.001	798.619 ± 118.813	698.859 ± 143.445	<0.001
Chao	758.083 ± 112.963	831.674 ± 118.627	<0.001	810.343 ± 126.667	704.223 ± 154.085	<0.001

### 3.4 Functional profiles of gut microbiota predicted by PICRUSt2

**FIGURE 7:**
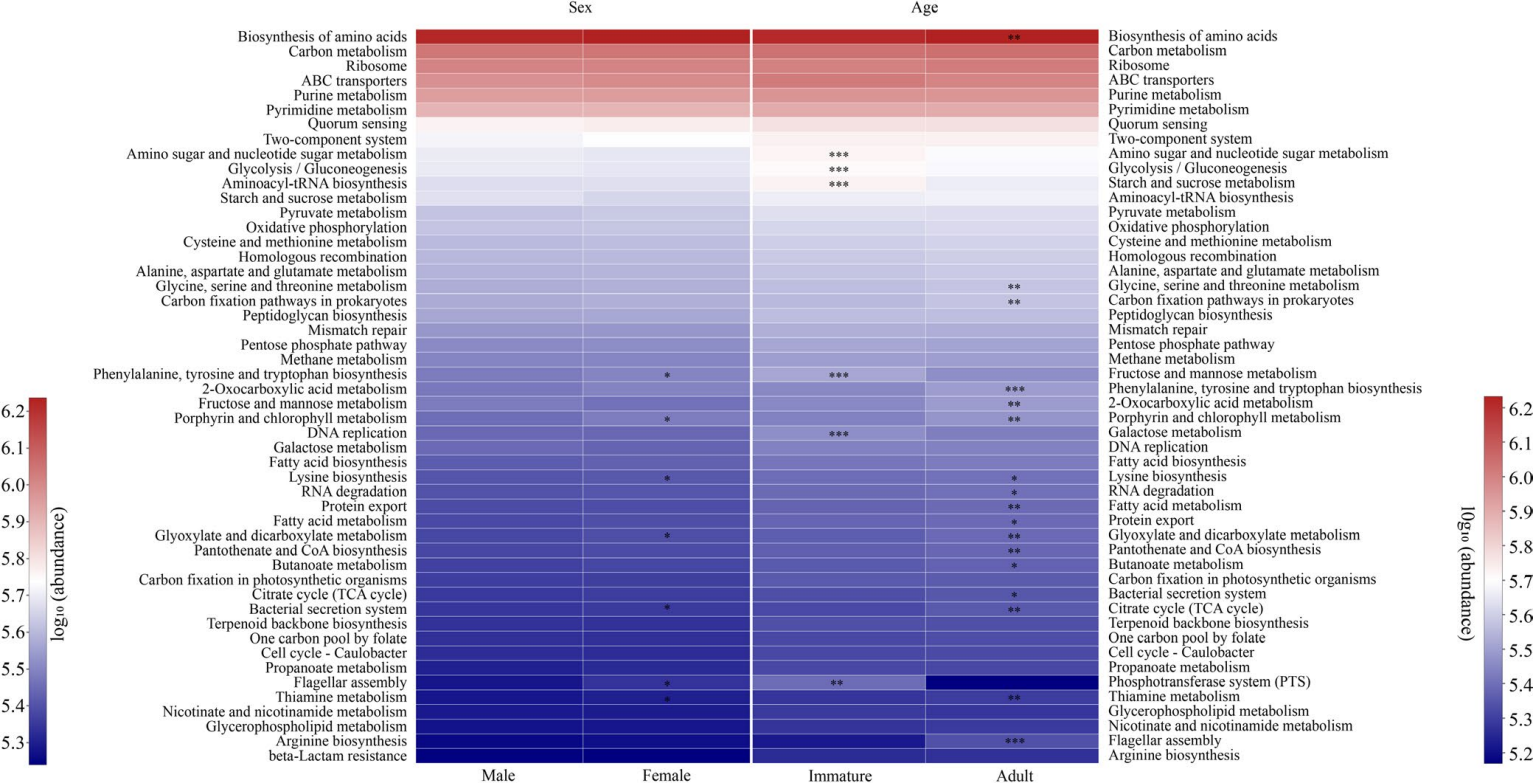
Differences in the functional profiles in pathway level 3 of the gut microbiota in sex and age. The *p*‐value is represented by asterisks. Significant difference 0.01 < *p* < 0.05 is marked as “*”, 0.001 < *p* < 0.01 is marked as “**”, and *p* < 0.001 is marked as “***”

Pathways associated with phenylalanine, tyrosine, and tryptophan biosynthesis; porphyrin and chlorophyll metabolism; lysine biosynthesis; glyoxylate and dicarboxylate metabolism; bacterial secretion system; flagellar assembly; and thiamine metabolism were significantly richer in females than in males, whereas no metabolic pathways in level 3 were found with greater abundance in males than in females (Figure 7). Based on age, pathways associated with amino sugar and nucleotide sugar metabolism, glycolysis/gluconeogenesis, and starch and sucrose metabolism were significantly richer in immatures compared with adults. However, significant enrichment in biosynthesis of amino acids, glycine, serine and threonine metabolism, carbon fixation pathways in prokaryotes was detected in adults (Figure 7).

## 4 DISCUSSION

In this study, the gut microbiota diversity, abundance, and function of rhesus macaques inhabiting limestone forest differed by sex and age. Specifically, female rhesus macaques had higher gut microbiota diversity and more biosynthesis and metabolism pathways than male. In addition, adult rhesus macaques had a higher diversity of gut microbiota, richer norank *Ruminococcaceae* and norank Clostridiales, and more functional metabolic pathways than immatures. These differences could be associated with sex‐ and age‐specific differences in diet and/or differences in hormone production.

### 4.1 Effects of sex on gut microbiota

Our results showed that female rhesus macaques had higher alpha diversity and richness of their gut microbiota than males. These results were consistent with our prediction 1. Similar results have been found in several primates species, including Verreaux's sifakas (*P. verreauxi*) (Koch et al., 2017), western lowland gorillas (*G*. *gorilla gorilla*) (Pafčo et al., 2019), and humans (Sinha et al., 2019). Considering the requirement for higher nutritional and/or energetic demands caused by reproduction (Dunbar et al., 2009; Koch et al., 2017; Li et al., 2014; O'Mara & Hickey, 2014), female primates might be expected to have higher dietary diversity, based on a behavioral strategy to supplement nutritional needs (Dunbar et al., 2009; Li et al., 2014). Sex differences in feeding behavior would result in distinct dietary substrates for gut microbiota and exert different selective pressures on the gut microecosystem, facilitating the acquisition or evolution of new microbial communities and changes in digestive efficiency and nutrient absorption in the host (David et al., 2014). Higher species diversity in a microecosystem indicates increased functional diversity and redundancy (Moya & Ferrer, 2016). Thus, increased gut microbial diversity in females suggests that the gut microbiota in females may have more diverse functions, facilitating digestion and metabolism. This is supported by differences in functional profiles, with enriched biosynthesis and metabolic pathways in the female gut microbiota compared with males (Figure 7).

In addition, it is noteworthy that the abundance of bacteria belonging to the phylum Proteobacteria in female rhesus macaques was higher than that in males, which could reflect physiological differences. Most bacteria belonging to the phylum Proteobacteria have been shown to induce inflammation in humans (Mukhopadhya et al., 2012). Females appear to be more susceptible to pathogenic bacteria while improving their ability to absorb nutrients and energy, as documented in black howler monkeys (*A*. *pigra*) (Amato et al., 2014) and humans (Koren et al., 2012). Interestingly, androgens play a role in protecting host health (Yurkovetskiy et al., 2013). When gut microbiota was transplanted from male mice into female mice, females showed a reduction in islet inflammation and autoantibody production as testosterone levels increased (Markle et al., 2013). Therefore, sex differences in the diversity and composition of gut microbiota in rhesus monkeys might have been driven, in part, by differences in sex steroid hormone production.

### 4.2 Effects of age on gut microbiota

Adult rhesus macaques showed higher alpha diversity of their gut microbiota and a higher abundance of norank *Ruminococcaceae* and norank Clostridiales, compared to immatures, which supports prediction 2. These differences might be associated with age‐related differences in diet. In general, the digestive system and gut microbiota develop with age (Derrien et al., 2019). In addition, the dietary composition of adults is more complex and more indigestible than that of immatures who are limited by mobility or hunting skills (Kashtanova et al., 2016; Schiel et al., 2010). Therefore, the increased gut microbiota diversity in adult rhesus macaques might correspond to their relatively higher dietary diversity. Higher gut microbiota diversity might provide adults with higher functional redundancy, contributing to a more stable gut microecosystem than that in immatures (Lozupone et al., 2012; Tian et al., 2020). In this regard, taxa such as *Ruminococcaceae* and Clostridiales have been considered specialists in the degradation of complex plant materials (Koeck et al., 2014; La Reau & Suen, 2018). A higher abundance of *Ruminococcaceae* and Clostridiales in adult rhesus macaques also suggests that the macaques mainly relied on these two microbial taxa to enhance their digestion of complex cellulose. Additionally, fiber‐enriched diets also are associated with a reduction in the Firmicutes/Bacteroidetes ratio (De Filippo et al., 2010). Despite their relatively simple dietary structure, immature macaques had a higher ratio of Firmicutes/Bacteroidetes than adults, which may be attributed to the need for increased nutrient absorption during their growth and development. Vulnerable groups such as immatures and females may increase the nutritional harvest from their gut microbiota to compensate for growth and reproduction (Amato et al., 2014). Furthermore, the abundance of Proteobacteria was higher in adult rhesus macaques. Similarly, geriatric marmosets had higher Proteobacteria abundance than younger individuals, which was assumed to be related to the decline in immune function with age (Reveles et al., 2019). Thus, both nutritional requirements and age‐related physiological factors influence gut microbiota composition.

However, it must be acknowledged that the effects of the sex–age class on the community structure of gut microbiota were relatively weak. Overall, sex and age only explained 1.33% and 2.79% of the variation in community structure of the gut microbiota of rhesus macaques, respectively (Figure 6). This implies that other underlying determinants also influence the structure of the gut microbiota. This study preliminarily quantified the effects of sex–age class on the gut microbiota of rhesus macaques. Future studies should focus on the comprehensive effects of multiple factors, such as host genetics, sex–age class, dietary composition, and climate on the gut microbiota, offering a more comprehensive analysis of the association between the host and their symbiotic gut microbiota.

There are several limitations to our study. We have documented a general pattern of differences in the gut microbiota of limestone forest‐dwelling rhesus macaques based on sex and age class. Our results must be viewed with caution given the unequal sample sizes for each sex and age class. Moreover, due to the difficulty in following the macaques across an entire day and collecting fecal samples in the karst forest, the effects of sex and age on the gut microbiota of macaques require more controlled studies. In addition, we did not accurately estimate the effects of provisioned foods from park managers and visitors on their gut microbiota. Considering the fact that the sex and age effects might be overshadowed by other factors, additional studies should focus on the interactive influences of multiple factors such as morphology and anatomy and diet on the gut microbiota. This would provide a more comprehensive basis for understanding the evolutionary significance of the macaque gut microbiota.

In summary, sex and age were associated with differences in the diversity and relative abundance of gut microbiota in rhesus macaques. Female rhesus macaques had a higher diversity of gut microbiota and more biosynthesis and metabolism pathways than male individuals. Adult rhesus macaques had a higher diversity of gut microbiota, richer norank *Ruminococcaceae* and norank Clostridiales, and more functional metabolic pathways than immatures. These differences could be linked to age‐ and sex‐specific differences in nutrient requirements and hormone levels, highlighting the effects of age and sex on the structure and function of the gut microbiota, as well as the need to consider physiological traits when conducting gut microbiota studies.

## CONFLICT OF INTEREST

The authors declare no conflict of interest.

## AUTHOR CONTRIBUTIONS

**Yuhui Li:** Formal analysis (equal); Investigation (equal); Writing‐original draft (lead); Writing‐review & editing (supporting). **Ting Chen:** Formal analysis (equal); Investigation (equal). **Youbang Li:** Funding acquisition (supporting); Writing‐review & editing (equal). **Yin Tang:** Investigation (equal); Project administration (equal); Writing‐original draft (equal); Writing‐review & editing (equal). **Zhonghao Huang:** Conceptualization (lead); Formal analysis (equal); Funding acquisition (lead); Methodology (lead); Project administration (equal); Supervision (lead); Writing‐review & editing (equal).

## Data Availability

All data are available in the open Figshare repository, and the link to the data is https://doi.org/10.6084/m9.figshare.14453421.v1

## APPENDIX A

**TABLE A1:** Differences in the relative abundance of gut microbiota (at the phylum level) among sex and age classes in rhesus macaques

Species name	Female‐Mean (%)	Female‐*SD* (%)	Male‐Mean (%)	Male‐*SD* (%)	Corrected *p*‐value	Adult‐Mean (%)	Adult‐*SD* (%)	Immature‐Mean (%)	Immature‐*SD* (%)	Corrected *p* value
p__Firmicutes	58.29	14.02	55.09	18.47	0.325	58.07	15.87	62.39	18.62	0.355
p__Bacteroidetes	31.55	13.29	35.73	18.39	0.325	31.50	15.20	30.15	18.95	0.578
p__Proteobacteria	4.72	4.92	3.08	5.01	0.023	4.55	5.33	2.12	2.46	0.009
p__Actinobacteria	1.19	1.15	2.08	2.84	0.426	1.25	1.12	2.28	2.99	0.352
p__Spirochaetes	1.49	1.92	2.38	3.21	0.838	1.85	2.61	1.11	1.58	0.031
p__Cyanobacteria	0.78	0.80	0.52	0.69	0.021	0.98	1.96	0.97	2.35	0.135
p__Tenericutes	0.95	0.66	0.56	0.43	0.002	0.90	0.67	0.52	0.40	0.003
p__unclassified_k__norank_d__Bacteria	0.28	0.41	0.15	0.23	0.027	0.25	0.38	0.13	0.20	0.020
p__WPS‐2	0.29	0.57	0.14	0.18	0.838	0.24	0.48	0.12	0.35	0.157
p__Verrucomicrobia	0.24	0.45	0.12	0.23	0.028	0.21	0.40	0.09	0.26	<0.001
p__Fibrobacteres	0.09	0.16	0.07	0.10	0.725	0.09	0.15	0.04	0.06	0.020
p__unclassified_d__Unclassified	0.05	0.05	0.04	0.04	0.190	0.04	0.05	0.03	0.04	0.212
p__Lentisphaerae	0.04	0.11	0.02	0.05	0.325	0.03	0.10	0.02	0.06	0.336
p__Chlamydiae	0.02	0.05	0.02	0.04	0.758	0.01	0.09	0.02	0.08	0.971
p__Elusimicrobia	0.02	0.10	0.01	0.07	0.325	0.02	0.05	0.01	0.02	0.076
p__TM7	<0.01	<0.01	<0.01	<0.01	0.325	<0.01	<0.01	<0.01	0.01	0.578
p__[Thermi]	<0.01	<0.01	<0.01	<0.01	0.292	<0.01	<0.01	<0.01	0.01	0.073
p__Fusobacteria	<0.01	<0.01	<0.01	<0.01	0.838	<0.01	<0.01	<0.01	<0.01	0.440
p__Chloroflexi	<0.01	<0.01	<0.01	<0.01	0.292	<0.01	<0.01	<0.01	<0.01	0.034
p__Acidobacteria	<0.01	<0.01	<0.01	<0.01	0.886	<0.01	<0.01	<0.01	<0.01	0.157
p__Synergistetes	<0.01	<0.01	<0.01	<0.01	0.292	<0.01	<0.01	<0.01	<0.01	0.282
p__OD1	<0.01	<0.01	<0.01	<0.01	0.838	<0.01	<0.01	<0.01	<0.01	0.578
p__norank_d__BacteriaBacteroidetes	<0.01	<0.01	<0.01	<0.01	0.935	<0.01	<0.01	<0.01	<0.01	0.392
p__WS6	<0.01	<0.01	<0.01	<0.01	0.697	<0.01	<0.01	<0.01	<0.01	0.578
p__GN02						<0.01	<0.01	<0.01	<0.01	0.578
p__Planctomycetes						<0.01	<0.01	<0.01	<0.01	0.578

**TABLE A2:** Differences in the relative abundance of gut microbiota (at the genus level) in female and male rhesus macaques

Species name	Female		Male		Corrected *p* value
	Mean (%)	*SD* (%)	Mean (%)	*SD* (%)	
g__Prevotella	25.140	13.510	28.970	18.420	0.674
g__norank_f__Ruminococcaceae	9.835	4.475	8.848	4.339	0.596
g__Lactobacillus	4.472	9.130	5.329	9.095	0.564
g__norank_f__Clostridiaceae	3.254	4.975	3.833	5.298	0.724
g__Oscillospira	3.507	2.547	3.441	2.580	0.964
g__norank_f__Lachnospiraceae	3.541	2.093	2.822	1.645	0.341
g__unclassified_o__Clostridiales	3.289	1.296	2.543	1.280	0.064
g__Succinivibrio	3.669	4.758	2.055	4.864	0.064
g__norank_o__Clostridiales	2.754	2.408	2.178	1.788	0.341
g__Blautia	2.488	1.337	2.256	1.659	0.469
g__norank_f__S24‐7	2.337	1.601	2.338	2.678	0.469
g__Faecalibacterium	2.375	1.922	2.291	2.600	0.720
g__Coprococcus	2.586	1.543	1.820	1.289	0.064
g__unclassified_f__Ruminococcaceae	2.143	1.113	1.728	0.890	0.393
g__Treponema	1.366	1.875	2.278	3.167	0.821
g__Ruminococcus	1.859	0.991	1.617	1.052	0.462
g__Sarcina	1.806	3.431	1.663	3.772	0.724
g__Roseburia	1.624	1.247	1.806	1.812	0.816
g__unclassified_f__Lachnospiraceae	1.845	1.068	1.516	0.991	0.341
g__Enterococcus	0.709	3.033	2.502	8.246	0.365
g__norank_f__Peptostreptococcaceae	1.308	1.659	1.308	1.550	0.720
g__norank_o__Bacteroidales	1.153	1.214	1.379	1.610	0.964
g__Phascolarctobacterium	1.341	1.818	1.149	1.623	0.604
g__[Prevotella]	0.948	0.934	0.945	1.231	0.720
g__norank_f__Christensenellaceae	1.048	0.925	0.724	0.586	0.427
g__norank_f__Coriobacteriaceae	0.818	0.705	0.904	1.162	0.720
g__norank_o__RF39	0.866	0.563	0.524	0.401	0.047
g__unclassified_o__Bacteroidales_c__Bacteroidia	0.507	0.595	0.757	1.270	0.638
g__Bifidobacterium	0.230	0.803	1.003	2.392	0.644
g__norank_f__[Mogibacteriaceae]	0.662	0.365	0.488	0.307	0.145
g__Dialister	0.503	0.649	0.558	0.644	0.804
g__[Ruminococcus]	0.466	0.271	0.473	0.377	0.724
g__Catenibacterium	0.347	0.588	0.497	1.503	0.720
g__Clostridium	0.413	0.287	0.350	0.300	0.471
g__Lachnospira	0.348	0.392	0.332	0.474	0.736
g__Bulleidia	0.402	0.383	0.272	0.318	0.226
g__norank_o__Streptophyta	0.418	0.762	0.254	0.535	0.287
g__CF231	0.352	0.409	0.311	0.438	0.634
g__SMB53	0.354	0.654	0.293	0.406	0.644
g__YRC22	0.340	0.467	0.287	0.522	0.473
g__norank_o__YS2	0.355	0.328	0.264	0.396	0.194
g__norank_o__GMD14H09	0.255	0.921	0.355	0.856	0.720
g__norank_f__RF16	0.311	0.610	0.247	0.560	0.720
g__Dorea	0.264	0.121	0.280	0.211	0.564
g__Pediococcus	0.420	2.471	0.066	0.321	0.846
g__Anaerovibrio	0.271	0.457	0.200	0.317	0.638
g__Lactococcus	0.253	2.104	0.202	1.222	0.720
g__unclassified_k__norank_d__Bacteria	0.283	0.405	0.153	0.228	0.145
g__norank_f__[Paraprevotellaceae]	0.198	0.265	0.233	0.256	0.724
g__norank_p__WPS‐2	0.289	0.570	0.136	0.179	0.852
g__Megasphaera	0.185	0.413	0.209	0.491	0.757
g__Butyrivibrio	0.148	0.170	0.219	0.331	0.638
g__norank_f__RFP12	0.243	0.450	0.116	0.225	0.157
g__norank_f__Erysipelotrichaceae	0.178	0.219	0.124	0.172	0.287
g__norank_f__Veillonellaceae	0.160	0.259	0.136	0.317	0.564
g__norank_f__Rikenellaceae	0.130	0.154	0.154	0.262	0.720
g__norank_f__Enterobacteriaceae	0.096	0.687	0.170	0.734	0.761
g__unclassified_f__Clostridiaceae	0.122	0.165	0.123	0.171	0.871
g__Flexispira	0.103	0.142	0.142	0.330	0.837
g__unclassified_o__Lactobacillales	0.107	0.436	0.130	0.473	0.287
g__Bacillus	0.210	1.888	0.012	0.037	0.804
g__norank_f__Lactobacillaceae	0.155	0.754	0.038	0.258	0.469
g__norank_o__RF32	0.092	0.130	0.075	0.130	0.474
g__Fibrobacter	0.093	0.159	0.074	0.100	0.737
g__Desulfovibrio	0.104	0.175	0.058	0.065	0.474
g__Sphaerochaeta	0.083	0.090	0.058	0.065	0.478
g__Rummeliibacillus	0.035	0.283	0.105	0.628	0.812
g__unclassified_p__Firmicutes	0.050	0.164	0.087	0.207	0.393
g__Collinsella	0.057	0.073	0.067	0.109	0.898
g__norank_f__p‐2534‐18B5	0.060	0.094	0.050	0.085	0.564
g__Sutterella	0.055	0.109	0.050	0.064	0.964
g__Slackia	0.050	0.051	0.043	0.043	0.724
g__Parabacteroides	0.047	0.062	0.043	0.060	0.757
g__unclassified_f__Coriobacteriaceae	0.029	0.037	0.054	0.162	0.950
g__unclassified_d__Unclassified	0.048	0.046	0.035	0.041	0.385
g__unclassified_p__Proteobacteria	0.036	0.054	0.046	0.064	0.724
g__unclassified_c__Bacilli	0.024	0.102	0.058	0.194	0.845
g__Streptococcus	0.027	0.034	0.053	0.130	0.720
g__unclassified_f__Veillonellaceae	0.033	0.049	0.046	0.132	0.742
g__Ruminobacter	0.057	0.181	0.017	0.058	0.564
g__Leuconostoc	0.029	0.196	0.043	0.180	0.945
g__norank_o__Rickettsiales	0.040	0.047	0.030	0.035	0.504
g__Acinetobacter	0.069	0.636	<0.001	0.001	0.724
g__unclassified_f__Erysipelotrichaceae	0.023	0.030	0.041	0.068	0.852
g__RFN20	0.036	0.089	0.025	0.040	0.341
g__[Eubacterium]	0.027	0.025	0.032	0.042	0.821
g__norank_f__Leuconostocaceae	0.004	0.018	0.055	0.290	0.975
g__norank_o__ML615J‐28	0.039	0.253	0.009	0.021	0.939
g__p‐75‐a5	0.026	0.040	0.021	0.035	0.469
g__Brachyspira	0.019	0.028	0.022	0.038	0.996
g__unclassified_c__Betaproteobacteria	0.030	0.061	0.010	0.035	0.469
g__norank_f__R4‐45B	0.027	0.070	0.013	0.036	0.597
g__norank_o__M2PT2‐76	0.020	0.043	0.018	0.067	0.575
g__Oxalobacter	0.018	0.020	0.016	0.024	0.720
g__Mogibacterium	0.014	0.023	0.019	0.038	0.928
g__norank_f__Elusimicrobiaceae	0.017	0.045	0.016	0.037	0.762
g__unclassified_o__Bacillales	0.031	0.277	0.001	0.002	0.720
g__Candidatus_Rhabdochlamydia	0.017	0.103	0.013	0.075	0.564
g__unclassified_f__[Mogibacteriaceae]	0.015	0.028	0.014	0.031	0.767
g__Pseudomonas	0.020	0.115	0.008	0.025	0.975
g__L7A_E11	0.015	0.042	0.012	0.026	0.767
g__rc4‐4	0.015	0.019	0.010	0.013	0.473
g__Anaeroplasma	0.012	0.017	0.011	0.026	0.341
g__unclassified_c__Alphaproteobacteria	0.015	0.045	0.008	0.017	0.891
g__norank_f__Anaeroplasmataceae	0.014	0.020	0.008	0.012	0.390
g__Solibacillus	0.022	0.204	<0.001	<0.001	0.469
g__unclassified_c__Clostridia	0.014	0.016	0.008	0.012	0.303
g__norank_f__Victivallaceae	0.015	0.048	0.007	0.016	0.742
g__Anaerofustis	0.012	0.015	0.009	0.019	0.064
g__Dehalobacterium	0.010	0.014	0.009	0.012	0.856
g__Actinobacillus	0.012	0.032	0.007	0.015	0.638
g__Coprobacillus	0.009	0.015	0.010	0.019	0.804
g__Anaerostipes	0.012	0.028	0.007	0.011	0.794
g__norank_f__Streptococcaceae	<0.001	0.002	0.017	0.083	0.278
g__Butyricimonas	0.011	0.022	0.005	0.006	0.564
g__Tetragenococcus	0.001	0.007	0.014	0.047	0.469
g__unclassified_f__Prevotellaceae	0.007	0.007	0.007	0.008	0.841
g__norank_f__Bacillaceae	0.014	0.058	<0.001	0.001	0.564
g__unclassified_f__Planococcaceae	0.014	0.116	<0.001	0.001	0.720
g__Veillonella	0.008	0.018	0.006	0.010	0.720
g__norank_f__Dehalobacteriaceae	0.008	0.013	0.004	0.008	0.341
g__unclassified_f__Enterobacteriaceae	0.008	0.028	0.004	0.014	0.278
g__norank_c__Alphaproteobacteria	0.008	0.031	0.003	0.011	0.841
g__norank_f__Peptococcaceae	0.008	0.010	0.003	0.004	0.064
g__norank_o__Acholeplasmatales	0.009	0.039	0.001	0.008	0.478
g__unclassified_f__Pasteurellaceae	0.007	0.023	0.003	0.006	0.564
g__unclassified_p__Tenericutes	0.007	0.013	0.003	0.006	0.232
g__norank_f__mitochondria	0.004	0.009	0.006	0.016	0.720
g__unclassified_o__Rickettsiales	0.002	0.006	0.004	0.011	0.720
g__norank_f__Desulfovibrionaceae	0.003	0.006	0.002	0.004	0.804
g__Alloscardovia	0.001	0.005	0.003	0.011	0.638
g__Bacteroides_f__Bacteroidaceae	0.002	0.009	0.001	0.003	0.720
g__Weissella	<0.001	0.004	0.003	0.022	0.757
g__norank_f__Xenococcaceae	0.001	0.003	0.002	0.010	0.720
g__Actinomyces	0.002	0.008	0.001	0.002	0.564
g__unclassified_f__Bifidobacteriaceae	<0.001	0.002	0.002	0.009	0.139
g__unclassified_f__[Paraprevotellaceae]	0.002	0.003	0.001	0.002	0.341
g__Staphylococcus	0.001	0.004	0.002	0.004	0.347
g__norank_f__Flavobacteriaceae	0.002	0.023	0	0	0.720
g__unclassified_c__Gammaproteobacteria	0.002	0.004	0.001	0.002	0.462
g__Wautersiella	0.002	0.020	0	0	0.720
g__Bilophila	0.001	0.003	0.001	0.007	0.846
g__norank_c__TM7‐3	0.001	0.003	0.001	0.003	0.663
g__unclassified_p__Spirochaetes	0.001	0.004	0.001	0.002	0.794
g__Epulopiscium	0.001	0.002	0.001	0.005	0.841
g__unclassified_f__Rhizobiaceae	0.001	0.002	0.001	0.002	0.720
g__Rickettsiella	0.002	0.010	0	0	0.411
g__norank_f__Prevotellaceae	0.001	0.002	0.001	0.002	0.723
g__norank_f__[Cerasicoccaceae]	0.001	0.003	0.001	0.002	0.928
g__Rubellimicrobium	<0.001	0.001	0.001	0.005	0.637
g__norank_f__Gaiellaceae	0.001	0.002	<0.001	0.002	0.755
g__Rhodoplanes	0.001	0.003	<0.001	0.001	0.564
g__Carnobacterium	<0.001	0.002	0.001	0.004	0.760
g__unclassified_f__Gemellaceae	0.001	0.002	<0.001	0.002	0.898
g__norank_o__HA64	<0.001	0.001	0.001	0.002	0.852
g__Rhodococcus	0.001	0.003	<0.001	0.001	0.570
g__Leptotrichia	<0.001	0.002	<0.001	0.002	0.943
g__Halomonas	<0.001	0.001	0.001	0.003	0.720
g__Paracoccus	<0.001	0.001	<0.001	0.002	0.943
g__Sphingomonas	<0.001	0.001	0.001	0.002	0.587
g__Pseudonocardia	<0.001	0.002	<0.001	0.001	0.898
g__unclassified_c__Spirochaetes	<0.001	0.002	<0.001	0.003	0.723
g__unclassified_f__Comamonadaceae	0.001	0.008	0	0	0.720
g__Burkholderia	0.001	0.002	<0.001	0.001	0.393
g__Granulicatella	0.001	0.002	<0.001	0.001	0.638
g__Methyloversatilis	0.001	0.006	0	0	0.638
g__Neisseria	0.001	0.004	0	0	0.365
g__norank_f__AKIW874	<0.001	0.002	<0.001	0.001	0.930
g__Rhodobacter	<0.001	0.002	<0.001	0.002	0.762
g__Mitsuokella	0.001	0.005	0	0	0.570
g__Porphyromonas	0.001	0.002	<0.001	0.001	0.564
g__Methylobacterium	<0.001	0.001	<0.001	0.001	0.756
g__Rubrobacter	<0.001	0.001	<0.001	0.002	0.478
g__Salinivibrio	<0.001	0.003	<0.001	0.001	0.767
g__norank_o__Stramenopiles	<0.001	0.001	<0.001	0.001	0.736
g__Arthrobacter	<0.001	0.001	<0.001	0.002	0.638
g__unclassified_o__Burkholderiales	<0.001	0.001	<0.001	0.001	0.623
g__norank_o__JG30‐KF‐CM45	<0.001	0.001	<0.001	0.002	0.341
g__norank_o__Rhizobiales	<0.001	0.002	<0.001	0.001	0.604
g__unclassified_f__Oxalobacteraceae	<0.001	0.002	<0.001	0.001	0.720
g__norank_f__Propionibacteriaceae	<0.001	0.002	<0.001	0.001	0.943
g__Rothia	<0.001	0.001	<0.001	0.001	0.723
g__Haemophilus	<0.001	0.003	<0.001	0.001	0.812
g__norank_f__Mycoplasmataceae	<0.001	0.001	<0.001	0.001	0.943
g__Leucobacter	<0.001	0.001	<0.001	0.002	0.821
g__Truepera	<0.001	0.001	<0.001	0.001	0.341
g__unclassified_p__Bacteroidetes	<0.001	0.003	0	0	0.638
g__norank_f__EB1017	<0.001	0.001	<0.001	0.001	0.945
g__unclassified_o__Sphingomonadales	<0.001	0.001	<0.001	0.001	0.929
g__Balneimonas	<0.001	0.001	<0.001	0.001	0.757
g__norank_f__Acetobacteraceae	0	0	<0.001	0.001	0.168
g__Bradyrhizobium	<0.001	0.001	<0.001	0.001	0.674
g__1‐68	<0.001	0.002	<0.001	0.001	0.812
g__Corynebacterium	<0.001	0.001	<0.001	0.001	0.812
g__Anaerococcus	<0.001	0.001	<0.001	0.001	0.928
g__norank_f__Rhodospirillaceae	<0.001	0.001	<0.001	0.001	0.762
g__Serratia	<0.001	0.001	<0.001	0.001	0.928
g__Kaistobacter	<0.001	0.001	<0.001	0.001	0.928
g__Brachybacterium	<0.001	0.001	<0.001	0.002	0.821
g__norank_o__SHA‐98	<0.001	0.003	0	0	0.720
g__Arcobacter	<0.001	0.002	0	0	0.638
g__unclassified_f__Synergistaceae	<0.001	0.001	0	0	0.469
g__Arcanobacterium	<0.001	0.003	0	0	0.638
g__unclassified_f__Phyllobacteriaceae	<0.001	0.003	0	0	0.720
g__unclassified_f__Streptomycetaceae	<0.001	0.001	<0.001	0.001	0.720
g__norank_f__Rhizobiaceae	<0.001	0.001	<0.001	0.001	0.812
g__Cetobacterium	<0.001	0.001	<0.001	0.001	0.964
g__Thermomonas	0	0	<0.001	0.001	0.393
g__Campylobacter	<0.001	0.001	0	0	0.563
g__norank_f__Xanthomonadaceae	<0.001	0.001	<0.001	0.001	0.638
g__unclassified_o__Actinomycetales	<0.001	0.001	<0.001	0.001	0.638
g__unclassified_f__Xanthomonadaceae	<0.001	0.001	<0.001	0.001	0.638
g__Megamonas	<0.001	0.001	<0.001	0.001	0.638
g__norank_f__Ellin6075	<0.001	0.001	<0.001	0.001	0.638
g__unclassified_f__Fusobacteriaceae	<0.001	0.001	<0.001	0.001	0.638
g__Phormidium	<0.001	0.001	<0.001	0.001	0.638
g__Chryseobacterium	<0.001	0.002	0	0	0.720
g__Rhizobium	<0.001	0.001	0	0	0.563
g__Peptoniphilus	<0.001	0.001	<0.001	0.001	0.960
g__norank_f__Caulobacteraceae	<0.001	0.001	<0.001	0.001	0.960
g__Pseudoxanthomonas	<0.001	0.001	<0.001	0.001	0.821
g__Comamonas	<0.001	0.001	<0.001	0.001	0.821
g__Bacteroides_f__Bacteroidaceae_o__Bacteroidales	<0.001	0.001	<0.001	0.001	0.960
g__norank_f__Hyphomicrobiaceae	<0.001	0.001	<0.001	0.001	0.960
g__Brevibacterium	0	0	<0.001	0.001	0.393
g__Spirosoma	0	0	<0.001	0.001	0.564
g__Flavisolibacter	0	0	<0.001	0.001	0.564
g__Kocuria	0	0	<0.001	0.001	0.564
g__unclassified_f__Pseudanabaenaceae	0	0	<0.001	0.001	0.564
g__Pedomicrobium	0	0	<0.001	0.001	0.393
g__norank_c__OPB41	<0.001	0.001	0	0	0.570
g__norank_o__CW040	<0.001	0.001	0	0	0.638
g__norank_f__Oxalobacteraceae	<0.001	0.002	0	0	0.720
g__norank_c__ABY1	<0.001	0.002	0	0	0.720
g__Cupriavidus	<0.001	0.001	0	0	0.570
g__Rathayibacter	<0.001	0.002	0	0	0.720
g__norank_o__MBNT15	<0.001	0.001	<0.001	0.001	0.821
g__Akkermansia	<0.001	0.001	<0.001	0.001	0.821
g__Mycobacterium	<0.001	0.001	<0.001	0.001	0.821
g__norank_f__Beijerinckiaceae	<0.001	0.001	<0.001	0.001	0.821
g__Ochrobactrum	<0.001	0.001	<0.001	0.001	0.821
g__unclassified_f__Intrasporangiaceae	<0.001	0.001	0	0	0.638
g__norank_f__Halobacteroidaceae	<0.001	0.001	0	0	0.720
g__Allobaculum	<0.001	0.001	0	0	0.720
g__Arenimonas	<0.001	0.001	0	0	0.720
g__norank_o__PK29	<0.001	<0.001	0	0	0.638
g__Exiguobacterium	<0.001	<0.001	0	0	0.638
g__norank_c__SC72	<0.001	<0.001	0	0	0.720
g__DA101	0	0	<0.001	0.001	0.564
g__norank_o__Sphingomonadales	0	0	<0.001	0.001	0.564
g__norank_c__ZB2	0	0	<0.001	0.001	0.564
g__norank_f__Cytophagaceae	0	0	<0.001	0.001	0.564
g__Leptospira	0	0	<0.001	0.001	0.564
g__Nannocystis	0	0	<0.001	0.001	0.564
g__Paenibacillus	0	0	<0.001	0.001	0.564
g__Odoribacter	0	0	<0.001	0.001	0.564
g__Trichococcus	0	0	<0.001	0.001	0.564
g__Deinococcus	<0.001	0.001	0	0	0.720
g__norank_f__Actinomycetaceae	<0.001	0.001	0	0	0.720
g__Wohlfahrtiimonas	<0.001	0.001	0	0	0.720
g__norank_f__Methylobacteriaceae	<0.001	0.001	0	0	0.720
g__Sanguibacter	<0.001	0.001	0	0	0.720
g__norank_f__Frankiaceae	<0.001	0.001	0	0	0.720
g__Pedobacter	<0.001	0.001	0	0	0.720
g__norank_f__Anaerolinaceae	<0.001	0.001	0	0	0.720
g__norank_f__Nocardioidaceae	<0.001	0.001	0	0	0.720
g__Aeromicrobium	<0.001	0.001	0	0	0.720

**TABLE A3:** Differences in the relative abundance of gut microbiota (at the genus level) in adult and immature rhesus macaques

Species name	Adult		Immature		Corrected *p* value
	Mean (%)	*SD* (%)	Mean (%)	*SD* (%)	
g__Prevotella	25.050	15.120	24.790	18.120	0.887
g__norank_f__Ruminococcaceae	9.543	4.686	7.094	4.114	0.028
g__Lactobacillus	4.226	8.527	6.031	9.846	0.237
g__norank_f__Clostridiaceae	3.430	5.158	6.061	5.933	0.102
g__Enterococcus	1.464	6.107	6.697	13.850	<0.001
g__Oscillospira	3.509	2.619	2.461	2.227	0.081
g__Sarcina	2.443	5.712	3.330	5.166	0.231
g__norank_f__Lachnospiraceae	3.391	1.988	2.318	1.707	0.005
g__unclassified_o__Clostridiales	3.076	1.376	2.473	1.161	0.062
g__Blautia	2.299	1.430	2.618	2.165	0.973
g__Faecalibacterium	2.092	1.678	2.460	3.117	0.646
g__norank_o__Clostridiales	2.750	2.417	1.748	1.610	0.010
g__norank_f__S24‐7	2.262	1.851	2.134	2.481	0.218
g__Succinivibrio	3.571	5.154	0.768	1.806	<0.001
g__norank_f__Peptostreptococcaceae	1.380	1.692	2.883	4.069	0.180
g__Coprococcus	2.364	1.502	1.835	1.528	0.047
g__Ruminococcus	1.924	1.146	1.790	3.168	0.033
g__unclassified_f__Ruminococcaceae	2.035	1.093	1.305	0.970	0.004
g__Roseburia	1.616	1.491	1.620	1.700	0.588
g__unclassified_f__Lachnospiraceae	1.815	1.160	1.332	0.967	0.039
g__Treponema	1.727	2.573	1.057	1.553	0.076
g__norank_o__Bacteroidales	1.200	1.392	1.070	1.118	0.546
g__Phascolarctobacterium	1.332	1.841	0.613	1.050	0.047
g__norank_f__Coriobacteriaceae	0.801	0.681	1.028	1.356	0.811
g__[Prevotella]	0.880	1.003	0.766	1.059	0.195
g__norank_f__Christensenellaceae	0.994	0.878	0.518	0.493	0.007
g__norank_o__Streptophyta	0.629	1.878	0.745	2.291	0.650
g__norank_o__RF39	0.826	0.586	0.492	0.388	0.008
g__Bifidobacterium	0.300	0.818	1.009	2.498	0.650
g__Clostridium	0.499	0.626	0.647	1.445	0.229
g__norank_f__[Mogibacteriaceae]	0.614	0.357	0.524	0.386	0.155
g__unclassified_o__Bacteroidales_c__Bacteroidia	0.645	0.964	0.402	0.951	0.008
g__Dialister	0.458	0.618	0.516	0.644	0.960
g__[Ruminococcus]	0.461	0.385	0.419	0.350	0.646
g__SMB53	0.360	0.580	0.484	0.651	0.479
g__Catenibacterium	0.330	0.548	0.461	1.575	0.151
g__Lachnospira	0.340	0.411	0.327	0.482	0.565
g__Bulleidia	0.382	0.393	0.275	0.252	0.412
g__norank_o__YS2	0.348	0.353	0.215	0.338	0.016
g__Dorea	0.260	0.161	0.299	0.213	0.747
g__norank_o__GMD14H09	0.247	0.891	0.283	0.719	0.646
g__CF231	0.345	0.440	0.184	0.272	0.025
g__YRC22	0.331	0.493	0.189	0.365	0.047
g__Pediococcus	0.296	2.168	0.183	0.699	0.447
g__norank_f__Enterobacteriaceae	0.071	0.608	0.395	1.210	0.024
g__Lactococcus	0.334	2.053	0.114	0.436	0.104
g__norank_f__RF16	0.309	0.644	0.130	0.203	0.097
g__norank_f__[Paraprevotellaceae]	0.212	0.279	0.223	0.556	0.447
g__unclassified_o__Lactobacillales	0.064	0.236	0.369	0.752	0.001
g__unclassified_k__norank_d__Bacteria	0.249	0.378	0.133	0.196	0.030
g__Anaerovibrio	0.258	0.443	0.113	0.201	0.039
g__norank_p__WPS‐2	0.240	0.480	0.124	0.354	0.218
g__Butyrivibrio	0.166	0.207	0.174	0.292	0.878
g__unclassified_f__Clostridiaceae	0.142	0.215	0.185	0.223	0.465
g__norank_f__Erysipelotrichaceae	0.162	0.193	0.147	0.231	0.437
g__norank_f__RFP12	0.210	0.401	0.092	0.260	0.001
g__Megasphaera	0.202	0.478	0.096	0.178	0.175
g__Bacillus	0.167	1.677	0.127	0.770	0.832
g__unclassified_p__Firmicutes	0.076	0.243	0.189	0.284	0.006
g__norank_f__Rikenellaceae	0.135	0.206	0.111	0.141	0.859
g__Flexispira	0.114	0.160	0.122	0.350	0.072
g__norank_f__Lactobacillaceae	0.092	0.619	0.143	0.522	0.650
g__norank_f__Veillonellaceae	0.168	0.302	0.062	0.119	0.084
g__Rummeliibacillus	0.027	0.251	0.200	0.801	0.102
g__unclassified_c__Bacilli	0.056	0.240	0.153	0.272	0.001
g__Carnobacterium	0.001	0.009	0.204	1.331	0.039
g__Leuconostoc	0.015	0.156	0.173	0.628	0.024
g__Collinsella	0.058	0.073	0.089	0.119	0.115
g__norank_o__RF32	0.086	0.127	0.057	0.117	0.081
g__Fibrobacter	0.092	0.153	0.039	0.056	0.033
g__Desulfovibrio	0.095	0.159	0.035	0.058	0.001
g__Sphaerochaeta	0.080	0.087	0.036	0.050	0.007
g__norank_f__Leuconostocaceae	0.023	0.211	0.091	0.337	0.499
g__Acinetobacter	0.055	0.565	0.055	0.262	0.811
g__Streptococcus	0.031	0.060	0.077	0.190	0.878
g__Pseudomonas	0.020	0.104	0.084	0.274	0.201
g__unclassified_f__Coriobacteriaceae	0.033	0.038	0.068	0.176	0.918
g__Slackia	0.049	0.051	0.045	0.039	0.980
g__norank_f__p‐2534‐18B5	0.059	0.096	0.034	0.063	0.084
g__unclassified_f__Planococcaceae	0.022	0.137	0.070	0.457	0.629
g__Sutterella	0.051	0.099	0.037	0.066	0.254
g__Parabacteroides	0.042	0.058	0.045	0.062	0.791
g__unclassified_d__Unclassified	0.042	0.045	0.033	0.037	0.254
g__unclassified_f__Erysipelotrichaceae	0.023	0.032	0.044	0.070	0.565
g__unclassified_p__Proteobacteria	0.038	0.058	0.028	0.047	0.274
g__norank_o__Rickettsiales	0.037	0.044	0.029	0.038	0.261
g__Serratia	<0.001	0.001	0.063	0.420	0.432
g__Mogibacterium	0.017	0.027	0.042	0.096	0.372
g__Ruminobacter	0.048	0.164	0.011	0.037	0.039
g__p‐75‐a5	0.024	0.033	0.034	0.069	0.878
g__[Eubacterium]	0.026	0.025	0.030	0.043	0.878
g__unclassified_f__Veillonellaceae	0.040	0.096	0.015	0.029	0.058
g__RFN20	0.033	0.081	0.019	0.033	0.102
g__Solibacillus	0.037	0.258	0.015	0.087	0.948
g__Comamonas	<0.001	0.001	0.052	0.345	0.546
g__unclassified_o__Bacillales	0.025	0.246	0.017	0.111	0.929
g__Chryseobacterium	0.001	0.005	0.041	0.269	0.256
g__norank_o__ML615J‐28	0.032	0.225	0.006	0.012	0.666
g__norank_f__R4‐45B	0.021	0.057	0.015	0.058	0.412
g__Brachyspira	0.021	0.029	0.013	0.033	0.047
g__unclassified_c__Betaproteobacteria	0.028	0.059	0.004	0.012	0.020
g__Candidatus_Rhabdochlamydia	0.014	0.092	0.017	0.079	0.978
g__norank_o__M2PT2‐76	0.025	0.062	0.004	0.013	0.010
g__Trichococcus	<0.001	<0.001	0.028	0.184	0.102
g__unclassified_f__Enterobacteriaceae	0.015	0.089	0.013	0.047	0.271
g__Oxalobacter	0.018	0.023	0.009	0.013	0.139
g__norank_f__Elusimicrobiaceae	0.018	0.046	0.007	0.016	0.102
g__L7A_E11	0.015	0.041	0.009	0.013	0.565
g__Coprobacillus	0.009	0.017	0.013	0.021	0.586
g__norank_f__Anaeroplasmataceae	0.011	0.015	0.010	0.020	0.217
g__unclassified_f__[Mogibacteriaceae]	0.017	0.033	0.003	0.005	0.013
g__Actinobacillus	0.010	0.028	0.011	0.020	0.565
g__rc4‐4	0.014	0.018	0.006	0.011	0.055
g__Anaeroplasma	0.013	0.020	0.007	0.022	0.007
g__unclassified_c__Clostridia	0.012	0.015	0.007	0.011	0.067
g__Anaerostipes	0.013	0.032	0.006	0.010	0.218
g__Anaerofustis	0.011	0.015	0.007	0.018	0.034
g__unclassified_c__Alphaproteobacteria	0.014	0.041	0.004	0.008	0.141
g__norank_f__Streptococcaceae	0.005	0.054	0.012	0.041	0.013
g__Anaerovorax	0.005	0.050	0.012	0.058	0.296
g__norank_f__Victivallaceae	0.013	0.044	0.004	0.007	0.650
g__Dehalobacterium	0.010	0.013	0.007	0.012	0.097
g__Kocuria	0.001	0.005	0.015	0.094	0.061
g__Butyricimonas	0.010	0.020	0.005	0.007	0.267
g__unclassified_f__Prevotellaceae	0.007	0.008	0.006	0.007	0.384
g__norank_f__mitochondria	0.005	0.011	0.008	0.020	0.650
g__Tetragenococcus	0.004	0.023	0.009	0.036	0.666
g__Veillonella	0.006	0.016	0.006	0.011	0.859
g__Proteiniclasticum	0	0	0.012	0.079	0.254
g__norank_f__Bacillaceae	0.011	0.052	<0.001	0.002	0.650
g__unclassified_f__Pasteurellaceae	0.005	0.019	0.006	0.015	0.650
g__norank_f__Dehalobacteriaceae	0.007	0.012	0.003	0.005	0.026
g__norank_f__Peptococcaceae	0.007	0.010	0.002	0.004	0.024
g__norank_c__Alphaproteobacteria	0.006	0.028	0.003	0.010	0.254
g__norank_f__Xanthomonadaceae	<0.001	0.001	0.008	0.054	0.546
g__unclassified_p__Tenericutes	0.007	0.012	0.001	0.003	0.010
g__Sphingobacterium	0	0	0.008	0.052	0.254
g__norank_o__Acholeplasmatales	0.008	0.036	<0.001	0.001	0.180
g__Weissella	0.002	0.025	0.005	0.024	0.139
g__Facklamia	0	0	0.007	0.043	0.254
g__norank_f__Xenococcaceae	0.001	0.003	0.005	0.014	0.231
g__Alloscardovia	0.001	0.004	0.004	0.012	0.056
g__unclassified_o__Rickettsiales	0.002	0.006	0.003	0.010	0.668
g__Arthrobacter	0.003	0.023	0.002	0.011	0.133
g__Bacteroides_f__Bacteroidaceae	0.002	0.007	0.003	0.008	0.700
g__Paracoccus	<0.001	<0.011	0.004	0.013	0.039
g__Actinomyces	0.001	0.003	0.003	0.010	0.980
g__Leucobacter	<0.001	0.001	0.004	0.022	0.859
g__Anaerotruncus	0	0	0.004	0.023	0.102
g__norank_f__Desulfovibrionaceae	0.002	0.006	0.001	0.002	0.689
g__Rubellimicrobium	<0.001	0.001	0.003	0.010	0.006
g__unclassified_f__Bifidobacteriaceae	<0.001	0.002	0.003	0.009	0.034
g__norank_c__TM7‐3	0.001	0.002	0.002	0.005	0.546
g__norank_f__Flavobacteriaceae	0.002	0.020	0.001	0.006	0.650
g__Wautersiella	0.002	0.017	0.001	0.006	0.650
g__unclassified_f__Rhizobiaceae	0.001	0.002	0.002	0.004	0.139
g__Staphylococcus	0.001	0.004	0.001	0.004	0.356
g__Luteimonas	0.002	0.020	<0.001	0.002	0.296
g__unclassified_f__[Paraprevotellaceae]	0.002	0.003	0.001	0.002	0.141
g__Kaistobacter	<0.001	0.001	0.002	0.008	0.254
g__Planomicrobium	0	0	0.002	0.015	0.254
g__Rhodoplanes	<0.001	0.001	0.002	0.004	0.086
g__norank_f__Gaiellaceae	0.001	0.002	0.001	0.003	0.254
g__Sanguibacter	<0.001	0.001	0.002	0.010	0.918
g__unclassified_c__Gammaproteobacteria	0.001	0.003	<0.001	0.001	0.026
g__Deinococcus	<0.001	0.001	0.002	0.010	0.546
g__Brachybacterium	<0.001	0.001	0.001	0.010	0.906
g__Sphingomonas	<0.001	0.001	0.001	0.004	0.039
g__unclassified_p__Spirochaetes	0.01	0.004	<0.001	0.002	0.544
g__Epulopiscium	0.001	0.003	0.001	0.004	0.928
g__Sedimentibacter	0.001	0.009	<0.001	0.001	0.917
g__Bilophila	0.001	0.005	<0.001	0.002	0.650
g__norank_f__Prevotellaceae	0.001	0.002	0.001	0.002	0.666
g__Paenibacillus	<0.001	0.001	0.001	0.007	0.544
g__Rickettsiella	0.001	0.009	<0.001	0.001	0.565
g__Balneimonas	<0.001	0.001	0.001	0.004	0.057
g__norank_f__[Cerasicoccaceae]	0.001	0.002	0.001	0.003	0.878
g__Rhodococcus	0.001	0.002	0.001	0.002	0.906
g__Pseudonocardia	<0.001	0.002	0.001	0.002	0.531
g__unclassified_o__Actinomycetales	<0.001	0.001	0.001	<0.006	0.254
g__Rubrobacter	<0.001	0.001	0.001	0.003	0.012
g__unclassified_f__Gemellaceae	<0.001	0.002	0.001	0.002	0.803
g__norank_f__Caulobacteraceae	<0.001	0.003	0.001	0.003	0.442
g__norank_f__AKIW874	<0.001	0.002	0.001	0.002	0.386
g__Methylobacterium	0.001	0.002	<0.001	0.002	0.859
g__norank_o__JG30‐KF‐CM45	<0.001	0.001	0.001	0.003	0.057
g__unclassified_f__Intrasporangiaceae	<0.001	0.001	0.001	0.005	0.918
g__norank_o__Rhizobiales	<0.001	0.002	<0.001	0.002	0.940
g__norank_f__Sanguibacteraceae	0	0	0.001	0.006	0.254
g__Tissierella_Soehngenia	0	0	0.001	0.006	0.254
g__Truepera	<0.001	0.001	0.001	0.002	0.028
g__Halomonas	<0.001	0.001	0.001	0.003	0.878
g__unclassified_c__Spirochaetes	<0.001	0.002	<0.001	0.003	0.786
g__norank_o__HA64	<0.001	0.002	<0.001	0.002	0.663
g__Leptotrichia	<0.001	0.002	<0.001	0.002	0.774
g__Stenotrophomonas	0	0	0.001	0.005	0.254
g__Neisseria	0.001	0.004	<0.001	0.001	0.499
g__Porphyromonas	<0.001	0.002	<0.001	0.001	0.859
g__norank_f__EB1017	<0.001	0.001	0.001	0.001	0.214
g__Granulicatella	<0.001	0.002	<0.001	0.001	0.650
g__Bradyrhizobium	<0.001	0.001	<0.001	0.002	0.832
g__norank_f__Acetobacteraceae	<0.001	<0.001	0.001	0.002	0.026
g__Burkholderia	0.001	0.002	<0.001	0.001	0.276
g__norank_f__Propionibacteriaceae	<0.001	0.002	<0.001	0.001	0.703
g__unclassified_o__Sphingomonadales	<0.001	0.001	0.001	0.002	0.139
g__unclassified_f__Comamonadaceae	0.001	0.007	0	0	0.650
g__Rhodobacter	<0.001	0.002	<0.001	0.002	0.700
g__Anaerococcus	<0.001	0.001	0.001	0.001	0.058
g__Methyloversatilis	0.001	0.005	<0.001	<0.001	0.553
g__unclassified_f__Fusobacteriaceae	<0.001	0.001	0	0	0.139
g__unclassified_f__Paenibacillaceae	<0.001	0.002	<0.001	0.003	0.650
g__Corynebacterium	<0.001	0.001	<0.001	0.002	0.440
g__Rothia	<0.001	0.001	<0.001	0.002	0.432
g__norank_o__Sphingomonadales	0	0	0.001	0.002	0.039
g__Dietzia	0	0	0.001	0.003	0.254
g__norank_f__Cytophagaceae	0	0	<0.001	0.002	0.020
g__Mitsuokella	0.001	0.004	0	0	0.465
g__unclassified_f__Phyllobacteriaceae	<0.001	0.003	<0.001	0.001	0.550
g__Haemophilus	<0.001	0.003	0	0	0.296
g__Salinivibrio	<0.001	0.002	<0.001	0.001	0.906
g__norank_o__Stramenopiles	<0.001	0.001	<0.001	0.001	0.499
g__norank_f__Rhizobiaceae	<0.001	0.001	<0.001	0.001	0.878
g__norank_f__Mycoplasmataceae	<0.001	0.001	<0.001	0.001	0.878
g__Thermomonas	0	0	<0.001	0.002	0.039
g__norank_f__Jonesiaceae	0	0	<0.001	0.003	0.254
g__norank_f__Dermatophilaceae	0	0	<0.001	0.003	0.254
g__Phormidium	<0.001	0.001	<0.001	0.002	0.546
g__norank_f__Beijerinckiaceae	<0.001	0.001	<0.001	0.001	0.254
g__Arcobacter	<0.001	0.002	<0.001	0.001	0.650
g__unclassified_o__Burkholderiales	<0.001	0.001	<0.001	0.001	0.565
g__norank_f__Ellin6075	<0.001	<0.001	<0.001	0.001	0.139
g__norank_c__BD1‐5	<0.001	<0.001	<0.001	0.002	0.650
g__norank_f__Nocardioidaceae	<0.001	<0.001	<0.001	0.002	0.650
g__unclassified_f__Streptomycetaceae	<0.001	0.002	0	0	0.254
g__unclassified_p__Bacteroidetes	<0.001	0.003	0	0	0.553
g__unclassified_f__Oxalobacteraceae	<0.001	0.002	<0.001	<0.001	0.296
g__norank_f__Rhodospirillaceae	<0.001	0.001	<0.001	0.001	0.550
g__norank_o__PK29	<0.001	0.001	<0.001	0.001	0.686
g__norank_f__Oxalobacteraceae	<0.001	0.001	<0.001	0.001	0.296
g__norank_f__Sphingobacteriaceae	0	0	<0.001	0.002	0.254
g__norank_o__Chlorophyta	0	0	<0.001	0.002	0.102
g__Spirosoma	0	0	<0.001	0.002	0.102
g__Janthinobacterium	0	0	<0.001	0.002	0.254
g__Devosia	0	0	<0.001	0.001	0.039
g__Rathayibacter	0	0	<0.001	0.002	0.254
g__norank_f__Hyphomicrobiaceae	<0.001	0.001	<0.001	0.001	0.546
g__unclassified_f__Xanthomonadaceae	<0.001	0.001	<0.001	0.001	0.546
g__Cetobacterium	<0.001	0.001	<0.001	0.001	0.906
g__norank_o__SHA‐98	<0.001	0.003	0	0	0.650
g__1‐68	<0.001	0.002	0	0	0.381
g__unclassified_f__Synergistaceae	<0.001	0.001	0	0	0.296
g__Arcanobacterium	<0.001	0.002	0	0	0.553
g__Megamonas	<0.001	<0.001	<0.001	0.001	0.296
g__Pedobacter	<0.001	<0.001	<0.001	0.001	0.650
g__Campylobacter	<0.001	0.001	0	0	0.381
g__Flavisolibacter	0	0	<0.001	0.001	0.254
g__unclassified_f__Pseudanabaenaceae	<0.001	0.001	<0.001	0.001	0.254
g__Peptoniphilus	<0.001	0.001	<0.001	0.001	0.906
g__Pseudoxanthomonas	<0.001	0.001	<0.001	0.001	0.650
g__norank_f__Methylobacteriaceae	<0.001	0.001	<0.001	0.001	0.906
g__Pedomicrobium	<0.001	0.001	<0.001	0.001	0.906
g__Rhizobium	<0.001	0.001	0	0	0.381
g__norank_o__MBNT15	<0.001	<0.001	<0.001	0.001	0.650
g__Wohlfahrtiimonas	<0.001	<0.001	<0.001	0.001	0.650
g__norank_f__Gemmataceae	<0.001	<0.001	<0.001	0.001	0.650
g__Aeromicrobium	<0.001	<0.001	<0.001	0.001	0.650
g__Ochrobactrum	<0.001	<0.001	<0.001	0.001	0.650
g__Akkermansia	<0.001	0.001	0	0	0.465
g__Bacteroides_f__Bacteroidaceae_o__Bacteroidales	<0.001	0.001	0	0	0.465
g__norank_c__OPB41	<0.001	0.001	0	0	0.465
g__norank_o__CW040	<0.001	0.001	0	0	0.553
g__norank_c__ABY1	<0.001	0.001	0	0	0.650
g__Cupriavidus	<0.001	0.001	0	0	0.465
g__norank_f__Erythrobacteraceae	0	0	<0.001	0.001	0.254
g__norank_o__I025	0	0	<0.001	0.001	0.254
g__Aerococcus	0	0	<0.001	0.001	0.254
g__norank_c__ZB2	0	0	<0.001	0.001	0.254
g__norank_f__Actinomycetaceae	0	0	<0.001	0.001	0.254
g__norank_f__Methylocystaceae	0	0	<0.001	0.001	0.254
g__norank_o__Burkholderiales	0	0	<0.001	0.001	0.254
g__norank_f__Thermoactinomycetaceae	0	0	<0.001	0.001	0.254
g__Leptospira	0	0	<0.001	0.001	0.254
g__Nannocystis	0	0	<0.001	0.001	0.254
g__Odoribacter	0	0	<0.001	0.001	0.254
g__unclassified_p__Cyanobacteria	0	0	<0.001	0.001	0.254
g__unclassified_p__Chloroflexi	0	0	<0.001	0.001	0.254
g__DA101	<0.001	0.001	0	0	0.553
g__Helicobacter	<0.001	0.001	0	0	0.650
g__norank_c__SC72	<0.001	0.001	0	0	0.650
g__Mycobacterium	<0.001	0.001	0	0	0.553
g__norank_f__Halobacteroidaceae	<0.001	0.001	0	0	0.650
g__Allobaculum	<0.001	0.001	0	0	0.650
g__Arenimonas	<0.001	0.001	0	0	0.650
g__Brevibacterium	<0.001	0.001	0	0	0.553
g__norank_f__Frankiaceae	<0.001	0.001	0	0	0.553
g__Exiguobacterium	<0.001	0.001	0	0	0.553
g__norank_f__Anaerolinaceae	<0.001	<0.001	0	0	0.650
g__Cardiobacterium	<0.001	<0.001	0	0	0.650
